# Beyond the zebrafish: diverse fish species for modeling human disease

**DOI:** 10.1242/dmm.012245

**Published:** 2013-11-21

**Authors:** Manfred Schartl

**Affiliations:** Department Physiological Chemistry, Biocenter, University of Würzburg, and Comprehensive Cancer Center Mainfranken, University Clinic Würzburg, 97078 Würzburg, Germany.

**Keywords:** Fish model, Cancer, Evolutionary mutant model, Natural variation

## Abstract

In recent years, zebrafish, and to a lesser extent medaka, have become widely used small animal models for human diseases. These organisms have convincingly demonstrated the usefulness of fish for improving our understanding of the molecular and cellular mechanisms leading to pathological conditions, and for the development of new diagnostic and therapeutic tools. Despite the usefulness of zebrafish and medaka in the investigation of a wide spectrum of traits, there is evidence to suggest that other fish species could be better suited for more targeted questions. With the emergence of new, improved sequencing technologies that enable genomic resources to be generated with increasing efficiency and speed, the potential of non-mainstream fish species as disease models can now be explored. A key feature of these fish species is that the pathological condition that they model is often related to specific evolutionary adaptations. By exploring these adaptations, new disease-causing and disease-modifier genes might be identified; thus, diverse fish species could be exploited to better understand the complexity of disease processes. In addition, non-mainstream fish models could allow us to study the impact of environmental factors, as well as genetic variation, on complex disease phenotypes. This Review will discuss the opportunities that such fish models offer for current and future biomedical research.

## Introduction

The usefulness of biological models in providing insight into disease mechanisms, diagnostics and treatment is undisputable. Indeed, the list of ground-breaking insights into human diseases and highly efficient drugs developed based on animal studies is ever-growing. Although basic biomedical research relies on a variety of biological model organisms, clinicians more closely involved in applied biomedicine tend to trust the models that are most similar physiologically to humans. This has given mammals, particularly rodents, an undisputed supreme position as a model system for human disease, dating back to the 1920s. The laboratory mouse has become the most widely used model principally owing to the ease by which it can be genetically modified.

**Fig. 1.**
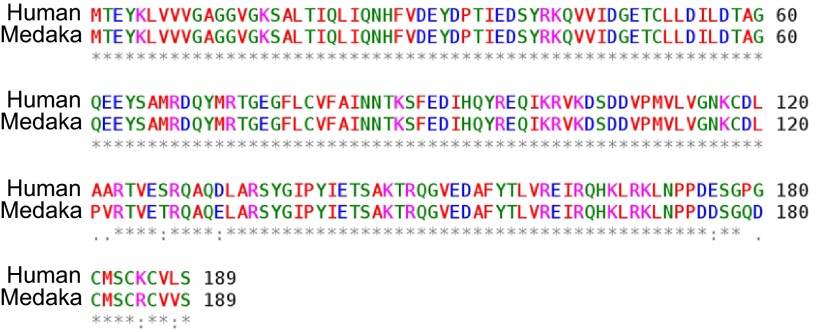
**Sequence comparison of the human and fish (medaka) HRAS proteins**. Below the alignment, identical amino acids are represented by an asterisk (*), a semi-conservative change is indicated by a dot (.) and a conservative change is indicated by a colon (:). The most frequent mutations in the STPase enzymatic active site of the protein in human cancers are found at amino acid positions 12 and 61.

More recently, there has also been an increasing appreciation of fish as human disease models. Admittedly, your guest in a restaurant looks distinct from the fish on his/her plate; however, scientists note only minor differences at the level of biological organization. Although fish diverged from humans more than 400-million years ago, there are enough commonalities to justify conducting research that is relevant to humans in these animals. At the molecular level, there are very few differences. For instance, the human *HRAS* gene, which is one of the most frequently mutated genes in cancer, shares over 95% identity with the corresponding gene in medaka. The few amino acid differences cluster in the carboxy terminus, far away from the enzymatic core of the protein, in which the human oncogenic mutations are found (Fig. 1). For biomedical research, fish provide a number of exceptional advantages: laboratory fish models are small and can be bred and maintained in large numbers easily and at low cost. They offer the opportunity to combine the analytical clarity of developmental biology with the power of genetics, and transgenic lines can be easily and quickly produced. Fish are also amenable to high-throughput approaches such as whole-genome mutagenesis or chemical library drug screens.

This Review does not discuss in detail the advantages, successes and promises of the zebrafish as the most prominent and most widely used fish model. Rather, it is an appeal that the research community does not put all of its eggs in one basket, and instead considers the opportunities that lie in the great biodiversity of different fish species. This plea has its origin in issues that were brought to light shortly after the whole human genome sequence was published, followed closely by the genome sequences of most model organisms, and an explosion of discovery relating to single-nucleotide polymorphisms, gene copy-number variation and quantitative trait loci (QTL). At the beginning of this ‘post-genomic’ era, several authors (Albertson et al., 2009; Bolker, 2012; Davis, 2004) pointed out that, despite their undisputable usefulness, the so-called ‘genetically domesticated’ model organisms have their limitations when it comes to the study of pleiotropy, multigenic inheritance, variable expressivity and variable penetrance of phenotypes. Also, natural environmental influences, which can profoundly modulate a phenotype, might be difficult or impossible to study in the indigenized, laboratory-adjusted classical models, because such environment-genotype interactions can be easily lost in selective breeding. Here, I first briefly discuss the mainstream genetically domesticated small aquarium fish models zebrafish and medaka. I then discuss why and how several other fish species are particularly advantageous for studying questions related to medical conditions that are more difficult to address in genetically domesticated models.

## Zebrafish and medaka: the major and mainstream models

The zebrafish is the canonical fish model for studying human disease. A large and steadily growing community relies heavily on this fish, courtesy of its excellent features, which have been highlighted in many reviews (e.g. Amatruda and Patton, 2008; Jing and Zon, 2011; Meijer and Spaink, 2011; Mione and Trede, 2010; Renshaw and Trede, 2012; Santoriello and Zon, 2012; Zon and Peterson, 2005). Although much less well-known and used only by a small, but steadily growing, community of researchers, the medaka *Oryzias latipes* (also known as the Japanese ricefish) can be regarded as a complementary model and is equivalent in many ways to the most common fish model (Wittbrodt et al., 2002). Like the similar-sized zebrafish, the medaka has a short generation time, is easy to breed in large numbers in the laboratory and produces transparent eggs, which make it easy to follow embryonic development.

One motivation to build up the medaka as a second laboratory fish model in parallel to the zebrafish is the complementarity argument. During their evolutionary history, teleost fish have undergone a whole-genome duplication leading to the situation of fish having two copies of many genes of which other vertebrates have only one (Amores et al., 1998; Braasch and Postlethwait, 2012; Meyer and Schartl, 1999; Postlethwait et al., 2000). This often manifests in altered gene expression patterns or protein functions, such that the complement of the expression domains or protein functions of both fish paralogs are equivalent to the single ortholog in other vertebrates. For instance, if a mouse gene is expressed in liver and kidney, one copy of the equivalent separate fish gene would be expressed in liver and the second copy would be expressed in kidney. This evolutionary process, which has been termed subfunction partitioning or subfunctionalization (Force et al., 1999), has far-reaching implications for functional studies of such genes. For example, when a mutation in mouse inactivates a gene that has a role in early development in addition to an adult organ-specific function, it becomes impossible to study the function of the adult organ in such mutants. In fish, both functions can be partitioned between duplicates, making it possible to separate both phenotypes. Thus, the teleost whole-genome duplication provides unique opportunities for studies on genes involved in disease development. Because there is a considerable amount of lineage-specific duplicate retention (meaning that one gene pair is retained and subfunctionalized in the zebrafish lineage, but not in medaka – and vice versa), the availability of more than one fish laboratory model will allow this situation to be fully exploited. In addition, gene function redundancy, which interferes with gene knockdown and knockout approaches, could differ between zebrafish and medaka, i.e. a paralogous gene resulting from the teleost whole-genome duplication event could take over the function of the mutagenized target gene, but, owing to differential duplicate retention, such redundancy might occur in one lineage but not in the other.

Both medaka and zebrafish provide a number of powerful laboratory tools. Transgenic fluorescent marker lines that allow these small aquarium fish models to be used for bioimaging have been developed in abundance. Systematic large-scale mutagenesis screens have led to a vast collection of mutants. The genomes of both fish are sequenced (Howe et al., 2013; Kasahara et al., 2007) and large genomic resources have been built up. For functional studies, a large toolbox exists in zebrafish and medaka: downregulation of gene expression during early development can be achieved by morpholinos, and gene knockout can be mediated by sophisticated genome-editing technologies such as zinc finger nucleases and TALENs. Furthermore, resource centers (NBRP for medaka, http://www.shigen.nig.ac.jp/medaka/; ZIRC for zebrafish, http://zebrafish.org/zirc/home/guide.php) have been established, from where mutants, natural and transgenic strains, and cDNA and genomic clones can be obtained.

A plethora of zebrafish and medaka disease models have been generated, making the zebrafish in particular the ‘laboratory mouse’ of fish models. Three common approaches have been used to generate these models. First, numerous mutational screens for developmental defect mutant embryos have been performed, and many of these brought to light phenotypes that resemble disease symptoms or affected genes that were already known to play a role in certain pathological conditions. Every mutant affecting the development of a certain cell type, tissue or organ can be used to better understand not only the physiology of normal development but also the pathological processes underlying congenital malformations, or chronic or degenerative diseases associated with those cells. Second, and in analogy to most mouse disease models, transgenic fish have been produced that express genes with known disease-related mutations. Third, a tool that is particularly easy to use in the translucent fast-developing embryos is gene knock-down with chemically modified short antisense oligonucleotides (‘morpholinos’) that are injected into embryos at the one-cell stage. Morpholinos can interfere with the expression of almost any gene of interest during embryonic and early larval development.

Overall, the existing fish models have provided outstanding opportunities for deciphering and understanding disease processes and identifying new molecular markers and therapeutic targets. Another key feature is the potential to perform high-throughput drug screens of chemical libraries using fish models of disease. Fish embryos and larvae can simply be immersed in medium containing the compounds and scored for phenotypic effects under a binocular microscope. Similarly, effects on disease markers can be assessed using high-throughput RNA *in situ* hybridization or protein immunostaining. Several such chemical screens, in which embryos that exhibit a certain disease-related phenotype are exposed in multiwell plates to tens of thousands of different compounds, have already been performed and led to the identification of promising candidate molecules (Santoriello and Zon, 2012).

Thus, the success of zebrafish and medaka as animal models for human diseases is well documented and widely accepted. Without questioning in any way the usefulness of the current approaches that exploit these fish, the present strategies have some notable limitations. In particular, zebrafish has been established primarily as an embryological or larval model for understanding vertebrate developmental biology. Many human diseases, however, occur in the adult or aging organism. This weakness becomes even more prominent when considering degenerative diseases. Studies aiming to better understand a neurodegenerative process might be more difficult to interpret when the disease is modeled in a mutant embryo that does not fully develop this neural structure.

**Fig. 2.**
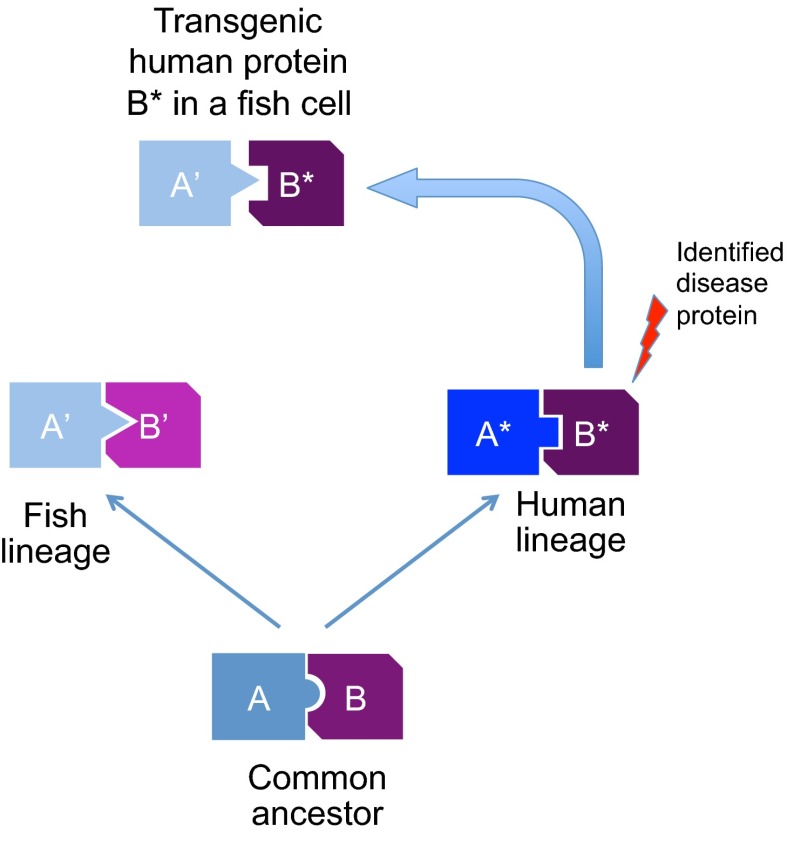
**Schematic representation of the process of protein divergence in different lineages after separation from a common ancestor**. Because interacting proteins evolve independently, the molecular structure of the interaction interface changes in both partners so that they still fit (i.e. coevolution occurs), but this will not necessarily affect the same amino acids. Eventually, protein A from one lineage is unable to properly interact with protein B from the other lineage (despite having fully retained its biochemical function, e.g. as an enzyme or G-protein). This process is known to evolutionary biologists as the Muller-Dobshansky-Bateson phenomenon. The divergence of interacting proteins can have an impact on studying the effect of a human-derived transgene in the non-human lineage.

In transgenic models that express a disease-associated gene, the human gene carrying the human mutation is generally used. This has in most cases been sufficient to produce a phenotype that satisfactorily mimics the human phenotype. However, even highly conserved proteins might have acquired subtle amino acid changes because of their fish- or human-lineage-specific coevolution with interacting or regulatory protein partners. Such interactive functions might escape detection during analysis (Fig. 2). This advocates for more subtle transgenic models in which, for example, the human disease mutation is introduced into the homologous fish gene.

## The concept of the ‘evolutionary mutant model’

Although zebrafish and medaka are the mainstream models, other fish models are now emerging from the backwaters of science, thanks to the perception that they are useful as ‘evolutionary mutant models’ for complex human diseases. This concept was first introduced by Craig Albertson, John Postlethwait and colleagues (Albertson et al., 2009). It is based on the awareness that evolution by adaptation to a specific environment has resulted in species or populations of many animal groups (including fish) that exhibit phenotypes closely resembling or mimicking human diseases. But are these other fish species – which are often experimentally less tractable than established models – useful for understanding human diseases? Moreover, can they be used to generate knowledge that cannot be more easily obtained from zebrafish and medaka?

Several lines of evidence support the idea that other fish species are valuable in disease research. For example, it has been argued that the mutations identified in forward genetic screens in zebrafish and medaka are biased. The screens conducted thus far have predominantly recovered mutant animals at the earliest developmental stage, when the gene displays an essential function. This often precludes studying disease phenotypes that develop later in life. By contrast, the disease-mimicking alterations in the evolutionary models manifest mostly in the adult or at least persist during adulthood.

Screens can also be biased towards mutations in the coding regions of genes, because the resulting phenotypes are more readily identified and show less variance. However, many mutations that cause a disease or increase disease susceptibility are found in the regulatory regions of genes (Horn et al., 2013; Huang et al., 2013; Lauderdale et al., 2000; Lettice et al., 2003; Loots et al., 2005; Sabherwal et al., 2007; Velagaleti et al., 2005). The genes that are involved in the phenotype that resembles the human condition in evolutionary models should, like the human genes, not be biased for coding mutations, and are expected to also harbor regulatory changes.

Mutagenesis screens often identify phenotypes that are more severe than the human disease they model. Traditional laboratory mutagenesis screens were set up to destroy genes; thus, the uncovered mutations strongly affect gene function and lead to severe phenotypes. For practical reasons, a stereotypical and strong phenotype in all individuals is desirable. Human diseases, however, can be ordered on a gradient from being very simple to extremely complex. At the low end are monogenic traits with little phenotypic variation in individuals that have the same disease allele. Many of the induced mutant models and transgenic models fall into this category. Other human diseases are caused by alleles of major effect, but the expression of such alleles is variable and considerably affected by modifier genes, i.e. the genetic background of the individual is important. At the high end of the spectrum are polygenic diseases such as cancer or cardiovascular disorders, for which the severity of the disease depends on the genotype of the patient at multiple loci, in addition to environmental influences. This complexity of human diseases, which is hard to capture experimentally and far from being understood, might be mirrored better by the evolutionary mutant models because the natural variation on the genes that correspond to human disease genes has created genetic diversity in a similar way as in humans.

In their seminal paper in 2009 (Albertson et al., 2009), Postlethwait and colleagues presented several examples for evolutionary mutant fish models. These will also be presented briefly here and complemented by additional natural models for human disease (summarized in Table 1). Generally speaking, the disease models can be categorized into different classes. One consists of evolutionary mutants in which the disease state is adaptive; the other encompasses fish that are afflicted with the same disease as humans, thus developing a true illness. A common feature across the disease models is allelic variation. Because of allelic variation, the same species of fish can provide completely independent models that fall into either the first or second class of disease models. Several examples of models within each of the two categories are described in the following sections.

**Table 1.**
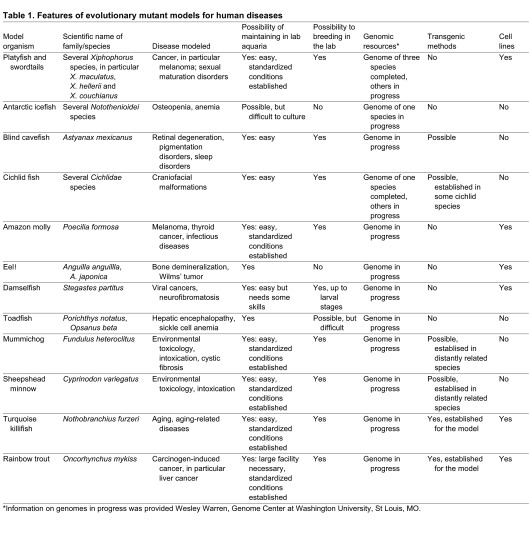
Features of evolutionary mutant models for human diseases

* Information on genomes in progress was provided Wesley Warren, Genome Center at Washington University, St Louis, MO.

### Models of adaptive disease phenotypes

#### Antarctic fish: models of osteoporosis and anemia

**Fig. 3.**
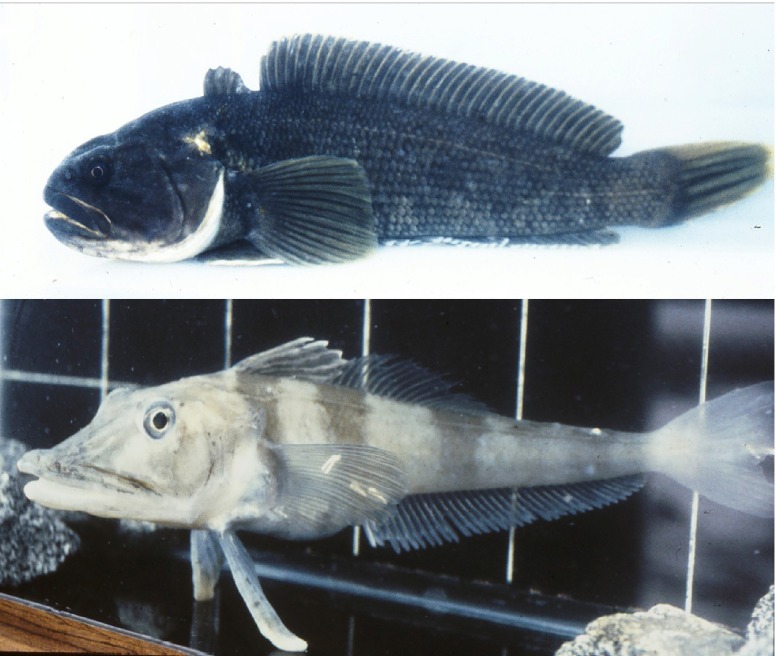
**Representative Antarctic notothenioid fish**. The Antarctic rockcod, *Notothenia coriiceps* (top), is red-blooded and possesses a robustly mineralized skeleton. The white-blooded icefish, *Chaenocephalus aceratus* (bottom), is profoundly anemic and osteopenic. Photographs provided by H. William Detrich (Northeastern University).

Paradigmatic examples for class I evolutionarily adapted models are found among the Antarctic fish (suborder Notothenioidei) (Fig. 3). In several species from multiple families of this suborder, changes in the musculoskeletal system occurred during evolution. To increase buoyancy, some of these fish display considerably decreased bone mineralization (Albertson et al., 2010; Kock, 2005). A reduction in bone mineral density is associated with a very common human condition known as osteopenia. It progresses frequently to the severe bone disease osteoporosis, which affects almost half of all women over 50 years of age as a result of changes in hormone levels. Osteoporosis is also seen in a substantial proportion of elderly men. The disease is characterized by bone microarchitecture deterioration and reduced bone mineral density, and these changes increase the risk of bone fracture. The identification and functional characterization of the notothenioid genes that are responsible for the natural osteopenia in these fish will contribute to a better understanding of the mechanisms that are involved in the regulation of bone mineral density and that are impaired in human bone diseases.

Additional buoyancy has been achieved in Antarctic fish by increasing lipid deposits in tissues and inner organs. In humans, lipidoses are a group of inherited metabolic diseases in which harmful levels of lipids accumulate in various organs, causing permanent cellular and tissue damage over time. Again, studies of notothenioid fish might help us to better understand the pathology of these human disorders.

The white-blooded icefish (Channichthyidae), a subtype of notothenioid fish, provide a model for human anemias. These are diseases characterized by a reduction in erythrocyte numbers or hemoglobin in the blood. The consequence is a potentially harmful insufficient supply of oxygen in the body. Well-known human conditions that cause anemia are thalassemias, sickle cell anemia and Fanconi anemia. The associated mutations affect hemoglobin or cellular functions, which are crucial for erythrocyte development or viability. Icefish usually have no erythrocytes, and do not use hemoglobin for oxygen transport. Icefish have special cardiovascular adaptations, which, combined with their relatively low metabolic rates and the high solubility of oxygen in the low-temperature water of their natural environment, ensure that their tissues receive an adequate supply of oxygen (Sidell and O’Brien, 2006). Comparing the white-blooded icefish with closely related species that produce hemoglobin and have erythrocytes has already led to the identification of a candidate gene that could be involved in determining the lack of erythrocytes in the former (Detrich and Yergeau, 2004), and has contributed to a better understanding of human red blood cell formation.

#### Cichlid fish: models of craniofacial developmental disorders

**Fig. 4.**
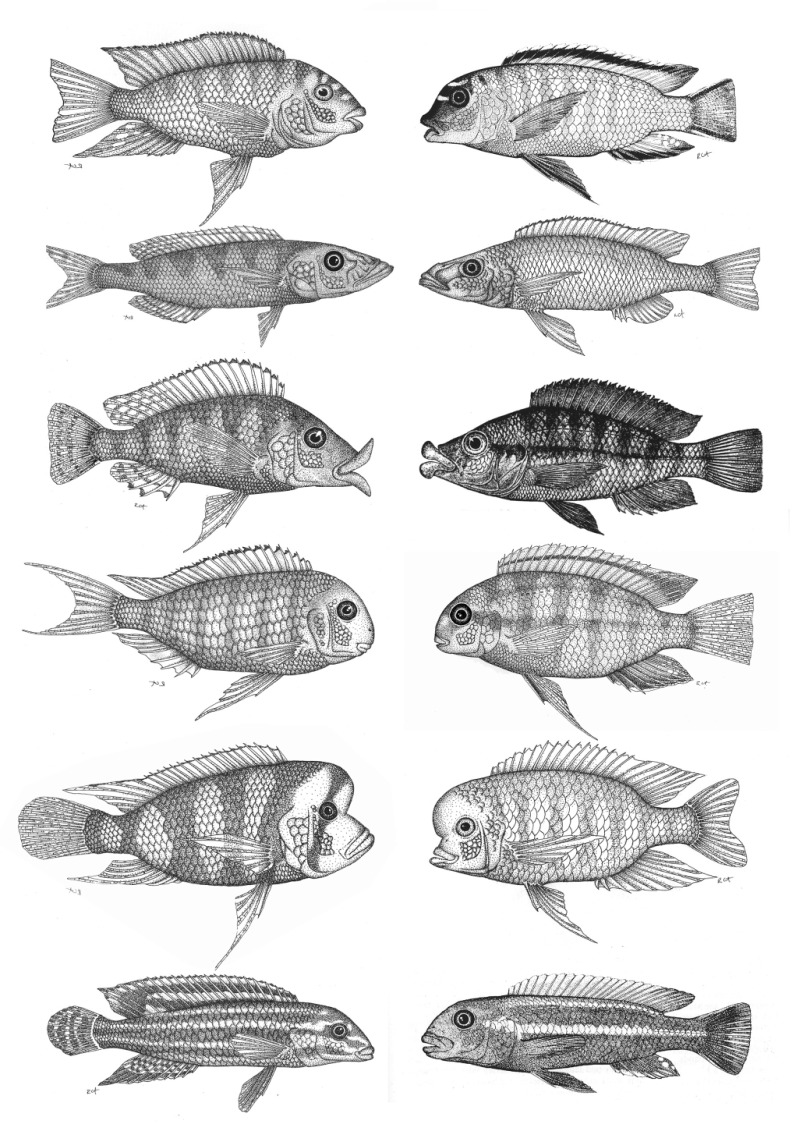
**Cichlid fish have evolved different craniofacial morphologies according to the diet to which the respective species has specialized**. This has led to differences in the morphology of the skull, and particularly the jaws. Interestingly, similar ecological adaptation has repeatedly led to the evolution of similar morphologies in different cichlid lineages. The picture shows similar ecomorphs from Lake Tanganyika (left) and Lake Malawi (right). Reproduced with permission (Albertson and Kocher, 2006), and outside the scope of the CC-BY license.

Freshwater fish from the family Cichlidae are a species-rich group of fish that display an outstanding variety of morphological and physiological adaptations to their specialized ecological niches. Differences in what they eat and where they feed have led to a great diversity in the morphology of the craniofacial skull, jaws and teeth in many cichlid fish species (Fig. 4). Studies on the differences in the development of structures that are responsible for these morphological specializations can contribute to an improved understanding of what goes wrong in medically important human craniofacial variation.

Models for craniofacial diseases that involve distortions in the construction of the face and jaw and dentition can be found among cichlids, and these have been proposed to reveal new targets for the prediction and treatment of human craniofacial disorders (Albertson et al., 2009). One study has shown that allelic differences affecting the *Patched-1* (*Ptc-1*) gene are responsible for adaptive variation in the shape of the lower jaw of Lake Malawi cichlid fish (Roberts et al., 2011). Patched-1 is a component of the sonic hedgehog signaling pathway, which has been shown to be involved in development of the mammalian upper jaw, and thus *PTCH1* could be a reasonable candidate gene for craniofacial disorders in humans (Cobourne and Green, 2012; Young et al., 2010). In line with this, a role for sonic hedgehog signaling in jaw development has been discovered in zebrafish (Eberhart et al., 2008), which nicely demonstrates the complementary nature of studies in non-mainstream model species.

Whereas the Antarctic fish and cichlid models described above are clearly the result of positive selection, the adaptive value of the disease-like phenotype in cave-dwelling fish and short-lived fish, which are discussed below, is less obvious, and might be the result of neutral or even regressive evolution.

#### Blind cavefish: models of retinal degeneration, albinism and sleep disorders

**Fig. 5.**
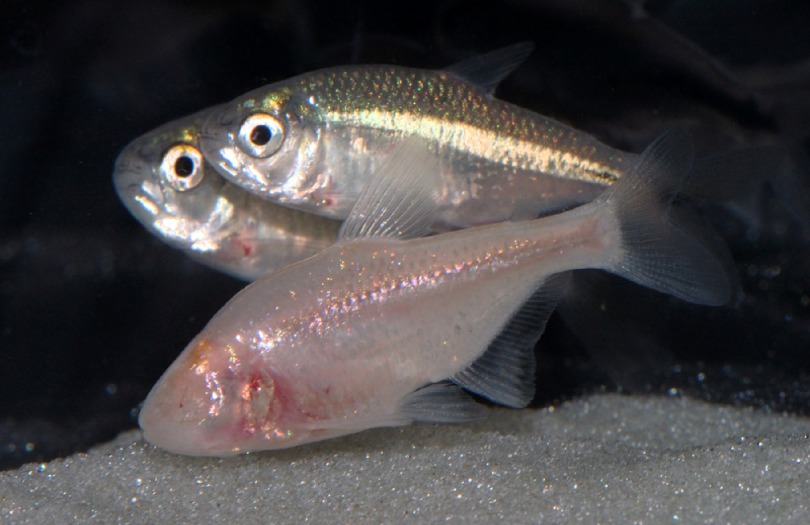
**Two epigean Mexican tetras (*Astyanax mexicanus*) and their eyeless unpigmented cave-dwelling relative**. Photo provided by Richard Borowsky (New York University).

The blind cavefish, *Astyanax mexicanus*, has traditionally been a source of fascination for evolutionary biologists, and its potential for illuminating disease phenotypes and their molecular basis has been recognized more recently. Like other animals that inhabit the lightless habitats of caves, *Astyanax* fish have, during their evolution, undergone degeneration of light-sensing organs and loss of pigmentation. In cavefish evolution, living in the dark has repeatedly and independently led to degenerated lenses and retinas (Fig. 5), resulting in a striking similarity to human retinal degradation disease symptoms.

The Mexican cavefish *Astyanax* is unique in that surface populations (living in light conditions) and cave populations co-exist and thus can be subjected to comparative studies (Borowsky and Wilkens, 2002; Gross, 2012; Strecker et al., 2003; Wilkens, 2010). Previous studies have indicated that differential regulation of certain eye development genes (e.g. *pax6*, *sonic hedgehog*) are linked to the eye degeneration process (Jeffery, 2009); however, these factors probably act as downstream mediators for so-far-unidentified mutant genes that initiate degeneration in these fish (Wilkens, 1988). QTL analysis has identified several loci that are involved in eye degeneration (O’Quin et al., 2013; Protas et al., 2007), but the genes encompassed by these loci are not yet known. Clearly, the full genome sequence of *Astyanax*, which is in the process of being deciphered (Wesley Warren, Washington University School of Medicine, personal communication), and detailed genetic maps are necessary to pinpoint the affected genes and clarify their role in eye development.

Human pigmentation disorders are sometimes regarded to be of ‘cosmetic’ relevance alone; however, some conditions such as albinism or vitiligo have been linked to severe psychological problems. Moreover, light skin is predisposed to skin cancer, and the absence of melanin in the retina can cause vision problems. A gene that is mutated in the melanin-less cavefish is *OCA2*, encoding an endoplasmic reticulum (ER)-localized transporter; the same gene has been shown to cause oculocutaneous albinism type II in humans (Protas et al., 2006). This provides a proof-of-principle example that mutations in the same gene can cause the same phenotype in human and fish.

The diurnal change of darkness and light is known to synchronize the periods of activity and sleep, and changes in the external zeitgeber seriously affects the ability to sleep, as everyone who has experienced jet lag will agree. In cave habitats, the daily environmental cues from light are absent, so the cave-dwelling *Astyanax mexicanus* populations have developed symptoms of sleep loss. Sleep disorders in humans include insomnia, advanced or delayed sleep phase syndrome, jet lag and shift-worker sleep disorder. Diverse neurotransmitter systems have been implicated in regulating sleep of mammals (Cirelli, 2013). In cavefish, physiological studies have revealed that blockade of β-adrenergic receptors with propranolol rescues the decreased-sleep phenotype. These findings indicate an involvement of β-adrenergic or NMDA receptor signaling (Duboué et al., 2012). The fact that different cave populations evolved the derived sleep loss independently through convergent mechanisms (Duboué et al., 2011) promises a wealth of information on possible molecular causes for the same phenotype of reduced sleep. Many different genes and pathways are expected to be involved in sleep regulation and it can be expected that, in the different cave populations, different genes have been mutated, each leading to disturbance of sleeping behavior.

#### Annual killifish: models for aging

Killifish are mostly small species of freshwater fish belonging to the order Cyprinodontiformes. Some killifish species have adapted to the severe ecological conditions provided by temporary ponds of the wet-dry tropical savannas. Their eggs and embryos survive the dry period in a stage of diapause in the soil, and hatch when the rainy season starts. The whole life cycle is then completed in the few months before the ponds dry out. Thus, most annual killifish have a short lifespan, even in the aquarium. This feature makes them superior models for aging research, because the other fish models under the same optimal conditions of aquarium care can live for several years, e.g. zebrafish reach an age of 6 years. In particular, the turquoise killifish *Nothobranchius furzeri* has been developed as an aging model (Terzibasi et al., 2007). Strains with different lifespans exist, the shortest being only 10 weeks and the longest 31 weeks from hatching to natural death. Aging fish show many general features of old organisms, including decreased fertility, cognitive decline, expression of age-related molecular markers and high morbidity. The availability of genotypes of *N. furzeri* that differ in average lifespan allows loci that control lifespan to be mapped by QTL analysis (Kirschner et al., 2012).

In *N. furzeri*, aging is connected to an increased risk of a number of diseases, including cancer, infectious diseases, circulation problems and neural degeneration. Why the aging organism displays this multimorbidity phenomenon is a question for which *N. furzeri* will provide a useful model.

#### Toadfish: models of hepatic encephalopathy and sickle cell anemia

The toadfish (Batrachoididae) are bottom dwellers that graze the sediment for worms, crustaceans, mollusks and other fish. Being slow movers and poor swimmers, they have evolved multiple strategies to hide from predators. One is to limit chemosensory signals, which is accomplished by the release of large amounts of urea to mask the scent of the toadfish. The excessive production of urea and a rather unique nitrogen excretion pattern makes toadfish extremely resistant to ammonia challenge, which is a threat to human health. Thus, toadfish, and in particular one well-studied species, the plain midshipman *Porichthys notatus*, have been proposed as a model for a human disease known as hepatic or portosystemic encephalopathy, which is due to liver failure (Walsh et al., 2008). The malfunction of the liver leads to confusion, altered levels of consciousness, coma and ultimately to death. One main cause of hepatic encephalopathy is excessive nitrogen load, e.g. following renal failure or due to inborn errors of the urea cycle.

Toadfish could also provide models for sickle cell anemia. A point mutation in human β-globin leads to the formation of a malfunctional hemoglobin known as HbS, which aggregates under low-oxygen conditions and consequently distorts erythrocytes to a sickle shape. Because these mutant cells confer a key adaptive advantage, namely resistance to malaria, the mutation is one of the most prevalent human inherited disorders. The toadfish hemoglobin behaves similar to the mutant HbS under hypoxia and induces a sickle-cell-like trait (Hárosi et al., 1998). The ordered aggregates in the red blood cells are remarkably similar in their biophysical features to those in human erythrocytes that contain HbS. Therefore, toadfish hemoglobins provide a useful tool for studying sickling disorders and other protein-aggregation-related phenomena. Interestingly, bottom-dwelling toadfish are virtually sessile and do not necessarily experience oxygen shortage, so the potential adaptive value of this trait is unclear. The feature might have developed as a neutral character.

The relatively big, raptorial, marine batrachoid fish might intuitively not look like a very good laboratory model, but the hardy adults are readily maintained in many laboratories, and eggs and larvae are also easily accessible and available. A species of toadfish has even joined zebrafish, medaka and *Xiphophorus* as an experimental organism aboard a space shuttle, demonstrating its robustness.

### Medical condition models

Several fish models that develop the same disease as are seen in humans have been established. The condition leads to sickness in those individuals, which is different from the situation in the models described above where adaptations to the environment have resulted in disease-like phenotypes in healthy individuals. Some of these models also have additional features that are adaptive for the fish under natural conditions but mimic a human disease, in the same way as the evolutionary mutant models.

#### *Xiphophorus*: models of melanoma and disorders connected to development of sexual maturity

**Fig. 6.**
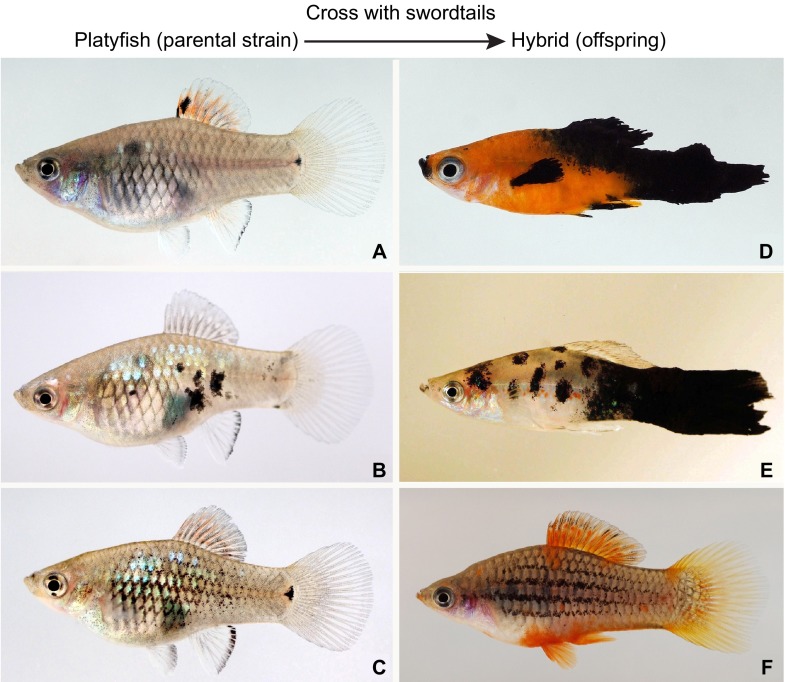
**Development of melanoma in hybrid offspring of platyfish and swordtails**. (A–C) Platyfish (*Xiphophorus maculatus*) with different pigmentation patterns are shown. (D,E) After crossing with swordtails (*X. hellerii*), offspring of A and B develop malignant melanoma. (A) This fish has pigment spots in the dorsal fin; (D) in the hybrids, which carry the *Sd* (spotted dorsal) allele of *Tu* and a closely linked tumor modifier, *mdl*, which determine the onset and compartment of pigment spot or melanoma formation, respectively, melanoma formation spreads out from this compartment. (B) This platyfish has black spots on the flanks determined by the *Sp* (spotted) allele of *Tu* and its linked *mdl*; (E) hybrids consequently develop melanoma from the body sides. Note that the dorsal fin of this fish is free of melanoma cells, which is different from the fish in D. (C,F) The fish in C carries the *Sr* (striped) allele of *Tu* along with *mdl*, which are not tumorigenic in the hybrids (F). The molecular basis of this is so far unknown.

*Xiphophorus* fish are one of the oldest animal models for cancer research (Heston, 1982) and the first to provide evidence that cancer has a genetic component. Early in the last century, it was found that certain hybrids of platyfish (*Xiphophorus maculatus*) and swordtails (*X. hellerii*) develop a highly malignant pigment cell tumor classified as melanoma (Gordon, 1927; Häussler, 1928; Kosswig, 1928). *Xiphophorus* is qualified as a true evolutionary mutant model because the malignant skin cancers develop from naturally occurring large pigment spots that are found in several species of this group of fish and have a function in kin recognition and mate choice (Fernandez and Bowser, 2010; Franck et al., 2001). Those spot patterns develop on the basis of an elaborate genetic interaction of a melanoma locus (*Tu*), whose activity is downregulated by a tumor suppressor locus (*R* or *Diff*) so that only large nevus-like spots occur, which never grow out to melanoma – with extremely rare exceptions (Schartl et al., 1995). Based on the fact that *Tu* and *R* are on different chromosomes, a crossing scheme can be set up to substitute the tumor-suppressor-containing chromosome pair by tumor-suppressor-free chromosomes of a closely related species that does not have the *Tu/R* system. In the hybrids, in the absence of *R*, the oncogene can manifest its deleterious function and highly malignant melanoma develop (Meierjohann and Schartl, 2006; Schartl, 2008) (Fig. 6). Besides this ‘classical’ crossing (the Gordon-Kosswig-Anders melanoma), a number of other melanoma models have been developed in *Xiphophorus* (Patton et al., 2010).

A candidate gene for the *R* locus is the *Xiphophorus* homolog of a cyclin-dependent kinase inhibitor, *CDKN2A/B*. Inactivation of the *CDKN2A* gene is a rare but highly penetrant risk factor for human familial melanoma (Meyle and Guldberg, 2009) and is also associated with many other cancers. The critical oncogene encoded by the *Tu* locus is *xmrk*, which encodes a mutationally activated version of the fish epidermal growth factor receptor (EGFR). Oncogenic signaling by EGFR and other related receptor tyrosine kinases is a hallmark of many tumors, including melanoma. In light of this, the *Xiphophorus* fish model has become a widely used system to study the genetics and molecular biology of melanoma formation. Studies on the tumor-inducing function of Xmrk in fish melanoma, complemented by analyses using established mouse and human cell culture systems in which *xmrk* was introduced as a transgene, have revealed important insights. For instance, the now well-established fact that the majority of human melanoma harbor mutations that confer constitutive activity to the Ras-Raf-MAPK signaling pathway was noted early on in the *Xiphophorus* system (Wellbrock and Schartl, 1999). Oncogenic signaling by Xmrk also activates the cytoplasmic Src-kinase Fyn (Wellbrock and Schartl, 1999; Wellbrock and Schartl, 2000), and the signal transducer and transcription factor Stat5 (Baudler et al., 1999; Morcinek et al., 2002), which prompted studies of these molecules in the context of human melanoma. These studies revealed roles of Fyn and Stat5 in proliferation, apoptosis, dedifferentiation and cell migration. Remarkably, Stat5 seemed to play an unexpected role in melanoma resistance to interferon α (IFNα) therapy (Wellbrock et al., 2005), which is a common problem in the treatment of human melanoma. Another Xmrk-regulated effector molecule, osteopontin (Geissinger et al., 2002), has been identified as a plasma marker for melanoma progression (Kluger et al., 2011; Zhou et al., 2005) and is now in use in the clinic. Crucial tasks for the near future are the firm identification and characterization of pathways involved in melanoma suppression.

An important aspect of this fish model is the fact that tumor-modifier genes have been defined via classical crossing experiments (Meierjohann and Schartl, 2006). Tumor-modifier genes are genetic determinants that are not involved in the primary events of neoplastic transformation as classical oncogenes or tumor-suppressor genes yet critically influence the course of the disease. They determine, for instance, whether the same cancer will be fatal for the individual or have a relatively mild progression. It will certainly be of high interest to unravel the molecular nature and the mode of action of these tumor-modifier genes from *Xiphophorus* and to extrapolate the findings to human homologs.

Other hybrid genotypes of *Xiphophorus* do not develop melanoma spontaneously and instead require UV irradiation (Mitchell et al., 2007). Studies on the radiation-induced tumors in fact have led to better definition of the hazardous UV spectrum and, in conjunction with data from mammalian models, have shown that prolonged chronic UV-A exposure does not contribute to melanomagenesis (Fernandez et al., 2012). In other crosses, the hybrid fish can be highly susceptible to develop other types of cancer following treatment with chemical carcinogens, allowing the study of genetic risk factors connected to susceptibility (Mitchell et al., 2007; Walter and Kazianis, 2001).

*Xiphophorus* fish also provide a model for diseases connected to impaired regulation of food uptake, energy balance and the onset of sexual maturity. In many species, males attain sexual maturity when they reach a certain stage of development that is defined by a particular body weight and size and correlates with age. Once this is accomplished, the males fully develop the secondary sex characters and cease to grow further in size. The period of puberty in *Xiphophorus* is genetically determined by a polymorphic locus, the *P*-locus, and leads to either small, early-maturing males or large, late-maturing males, or a continuum of intermediate size classes. This link between puberty and energy balance is intriguing from a medical point of view because it is known for instance that anorexia nervosa in young girls goes along with delayed puberty whereas early childhood obesity leads to premature menarche. The onset of puberty in *Xiphophorus* is determined by copy-number variation affecting the melanocortin 4 receptor at the *P*-locus (Lampert et al., 2010). This receptor is known to play a crucial role in the very complex network that controls food uptake and energy balance in humans, and mutations in this receptor are connected to obesity and early-onset puberty. The evolutionary model of genetically controlled puberty in *Xiphophorus* provides a novel tool to analyze this multicomponent system, which integrates signals not only from the hypothalamic melanocortin 4 receptor system but also from adipose tissue, via leptin, and thereby links the nutritional status to reproduction.

The *Xiphophorus* model is currently ahead, from a logistics point of view, of all the other evolutionary models because it offers a number of resources for the community. The genome is sequenced (Schartl et al., 2013) and available on the EMSEMBL genome server (http://uswest.ensembl.org/Xiphophorus_maculatus/Info/Index), cell lines have been developed, and a genetic stock center at the University of San Marcos (http://www.xiphophorus.txstate.edu/) provides inbred lines and strains derived from natural populations, as well as comprehensive information on protocols, genetic maps and reference databases.

#### Eels: models of physiological bone demineralization and childhood kidney cancer

Eels, members of the Anguilliformes order, are characterized by an elongated body axis. Understanding how changes in the embryonic program for the production of additional segments of the body (somites) that form cartilage, muscle and bone are brought about in eels could enable a better understanding of pathological changes that affect these structures in a variety of human disorders and diseases. Somitogenesis has been studied using a loss-of-function (i.e. mutagenesis) approach in zebrafish and medaka (genes were identified because interfering with their function led to loss of somites), but eels provide an opportunity to examine this process in a gain-of-function model (the identification of genes based on the formation of additional somites). Eels have also lost their pelvic fins, which are homologous structures to the hindlimbs of tetrapods (Burke and Rosa-Molinar, 2002). Deformations of legs affect several tens of thousands of humans every year, and the underlying developmental and molecular mechanisms are barely understood.

Eels are also known to undergo bone resorption in situations of physiological stress, for instance during fasting or during their extensive migrations for reproduction. Thyroid hormone has been shown to be involved in demineralization of bone in eels (Sbaihi et al., 2007), and an overproduction of the same hormone in humans can induce bone loss and osteoporosis. The cellular and molecular mechanisms of thyroid hormone action in bone remain, however, controversial (Nicholls et al., 2012). The eel therefore provides a complementary model to mammalian models for the study of osteoporosis in humans, and could contribute to a better understanding of the action of thyroid hormone and bone demineralization.

Last but not least, eels are a natural model for Wilms’ tumor (Masahito et al., 1992), a malignant kidney tumor that affects 1 in 10,000 children at early age. This tumor occurs ‘spontaneously’ with high prevalence in these fish in the wild. Such a natural model is not available elsewhere, and previous studies have relied on a somewhat complicated and highly engineered transgenic mouse model (Hu et al., 2011).

#### The Amazon molly: a clonal model

The Amazon molly, *Poecilia formosa*, stands out from all other vertebrate aquatic models by virtue of its unique feature of genetic clonality (Lampert and Schartl, 2008), which is a result of its unusual mode of reproduction. Females produce diploid eggs without meiosis; in response to sperm of males from related species, the eggs then undergo parthenogenesis. There is, however, no paternal genetic contribution and all offspring develop as clones of their maternal lineage. Many different clones exist in nature (Stöck et al., 2010). The proven true clonality of lines maintained in the lab allows researchers to perform experiments with animals that are genetically identical. This guarantees utmost reproducibility of independent studies, a stereotypical development of diseases or physiological processes under scrutiny and the generation of highly comparable biological material. Importantly, studies in a natural clonal vertebrate allow disease phenotypes and underlying mechanisms to be studied without the influence of the genetic makeup of the experimental animal. Such questions are generally barely understood and cannot be addressed in a similar way *in vivo* in any other vertebrate disease model. Thus far, the Amazon molly has been used for studies on melanomatous skin cancers (Schartl et al., 1997), thyroid cancer (Woodhead et al., 1984) and infectious diseases (Tobler and Schlupp, 2005), but it is hoped that a much broader spectrum of disease models can be developed.

#### Damselfish: models of cancers caused by viruses and of neurofibromatosis

**Fig. 7.**
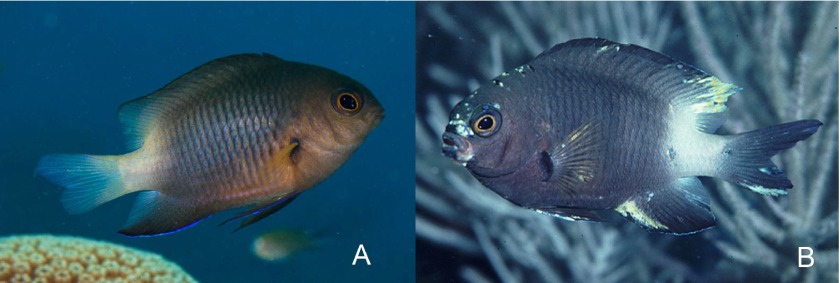
**Healthy and diseased bicolor damselfish (*Stegastes partitus*)**. (A) Healthy; (B) diseased. The diseased fish shows several pigmented lesions (chromatophoromas) and a non-pigmented neurofibroma in the corner of the mouth. Photos provided by Michael Schmale (University of Miami).

An estimated 15% of all human cancers worldwide might be attributed to viruses (Liao, 2006). The study of viruses that cause tumors in animals has contributed essential information to our understanding of the molecular biology of cancer genes. The majority of tumors caused by DNA viruses and retroviruses are papillomas, sarcomas, lymphomas and leukemias (Fan, 1994). Damselfish neurofibromatosis (DNF) is an example of a naturally occurring tumor affecting neuroectodermal cells, and is caused by an as-yet-unknown transmissible virus-like agent (Rahn et al., 2004). DNF is a disease that naturally affects bicolor damselfish *Stegastes partitus* (Fig. 7) on Florida reefs. It is characterized by the development of multiple neurofibromas, malignant peripheral nerve tumors and pigment-cell tumors (Schmale et al., 1983; Schmale et al., 1986). Its characterization is important because it could be representative of a potential new class of tumor-inducing-transmissible agents.

#### Mummichog and the sheepshead minnow (killifish): models for environmental toxicology

The disease models discussed so far can be used to obtain a better understanding of the mechanisms of disease development and to improve diagnosis and therapy. However, some fish that are afflicted by the same diseases as humans have evolved features that make them resistant to such diseases, in particular those evoked by toxicants from the environment. The mummichog *Fundulus heteroclitus* inhabits a wide range of mainly brackish, but also freshwater and marine, habitats all along the North American east coast from northern Florida to Canada. This fish shows an enormous plasticity and occupies diverse ecological niches. Some populations have evolved tolerance to hypoxia, extreme tolerance to high or low temperature and, most interestingly, tolerance to pollution coming from industrial, agricultural and municipal sources. Thus, this fish is potentially amenable to toxicogenomics analyses to gain insight into the processes of toxicant uptake, metabolism and toxicity. As a relatively small fish that can be maintained and bred in the laboratory, *Fundulus* has already served as a biological model in many other areas, including embryology, physiology, ecology and evolution (Burnett et al., 2007). Thus, its physiology, and molecular and developmental biology are well understood. From a human health standpoint, the mummichog has been used as a model for the study of physiological resilience and adaptations (Burnett et al., 2007). The adaptations to different salinity involves ion channels and transporters; thus, diseases or physiological processes in which such molecules are involved (e.g. osmoregulation, cystic fibrosis, cardiac physiology) could benefit from studies in *Fundulus*.

Inhabiting similar environments as the mummichog, but farther southwards, and belonging to the same order of fish (Cyprinodontiformes), the sheepshead minnow (*Cyprinodon variegatus*) is similarly adapted to fluctuating environmental conditions. Owing to their hardiness and the ease with which they can be maintained and bred in the laboratory, they have been commonly used in toxicology experiments (Hinton et al., 2005). Like the mummichog, this fish has been developed as a model for studies on xenobiotic exposure and human intoxication (Hinton et al., 2005).

#### Rainbow trout: models of liver cancer induction by environmental carcinogens

The rainbow trout (*Oncorhynchus mykiss*) is one of the oldest animal models for human cancer, in particular liver cancer. This species has a remarkable sensitivity to environmental carcinogens. In fact, the human liver carcinogen aflatoxin B_1_ produced by the common mold *Aspergillus* was first identified in 1960 when an epizootic outbreak of hepatoma was noted in trout hatcheries in California after fish had been fed with molded cottonseed flour (Jackson et al., 1968). The histopathology of trout liver cancer is strikingly similar to that in humans. As in humans, mutations in the *KRAS* oncogene are also common in trout liver cancer, and changes in the gene expression program in response to liver damage are comparable between the human and rainbow trout. After becoming an established model of liver cancer induction, these fish were used to identify many other environmental chemicals with a tumor-inducing or tumor-promoting effect. More recently, strategies were also developed for cancer chemoprevention (Williams, 2012). Dietary supplements were tested and some that efficiently inhibit liver cancer initiation following exposure to e.g. aflatoxin were identified. Trouts have key unique advantages in such studies; for instance, several tens of thousands of fish can be screened to determine dose-response relationships at levels orders of magnitude lower than is possible in rodents. In addition to being an economically important aquaculture species, a wealth of knowledge about trout biology, biochemistry, molecular biology and genetics exists. Although more tedious than in the small laboratory fish owing to the organism’s lengthy generation time, methods to create transgenic and knockout models have been established. Thus, it is possible and reasonable to develop other human disease models based on the rainbow trout, in particular other cancers.

## Conclusions and outlook

The value and advantages of the naturally occurring evolutionary mutant models can hardly be summarized in one sentence. It is clear that there are few unifying characteristics besides those that lie in the concept of the evolutionary mutant model itself. Each fish species reviewed here presents with only one or very few disease-related phenotypes. However, these offer the opportunity to ask questions about this particular disease in a different way than in the traditional ‘genetically domesticated’ models. Finding molecules, mechanisms and aberrations that are evolutionarily conserved between fish and humans gives a strong indication that they are truly relevant to a given disease. In addition, studying the mutant models in their natural habitats, or modeling certain aspects of their life history in the laboratory, enables assessment of environmental influences on the symptoms and course of diseases.

Also in the case of the classical fish disease models, zebrafish and medaka, researchers are starting to make use of wild-caught strains, which can be evaluated for evolution-driven mutations. In medaka, a number of highly inbred strains have been established, which are derived from different natural populations or laboratory fish stocks. This provides a unique genetic resource that can be used to study natural allelic variation of disease-modifier genes.

One example in which genetic variation that is fixed in a panel of inbred lines gives important information on disease-modifier genes is a transgenic melanoma model in medaka (Schartl et al., 2010). Here, the melanoma-inducing *xmrk* oncogene from *Xiphophorus* under control of a medaka promoter (from the pigment-cell-specific *mitfa* gene) is expressed specifically in pigment cells. As described earlier, *Xiphophorus* spontaneously develop malignant melanoma with high penetrance. Interestingly, when the same transgene is expressed in various inbred strains, depending on the genetic background, different types of tumor can result: highly invasive and metastasizing fatal melanoma from extracutaneous sites; much less life-threatening, large exophytic masses of tumors on the integument; or malignant melanoma of the uvea (Schartl et al., 2010). These distinct types display different gene expression signatures (Schartl et al., 2012), which can be ascribed to the presence of different tumor-modifier genes or allelic variation in the different strains. Whole-genome sequencing of these strains and a large panel (approximately 200) of near-isogenic strains established from a single wild population of medaka will provide a unique resource to identify disease-modifier genes and to analyze the interplay between natural genetic variation and disease (Spivakov et al., 2013).

There is a technological aspect that also contributes considerably to the renaissance of evolutionary mutant models. New sequencing technologies now enable full genomic resources to be produced at low cost, even by very small consortia, which will allow the study of disease processes in those models at the molecular level – an opportunity that until recently has existed only for a handful of carefully selected mainstream models. Consequently, the genomes and transcriptomes of several of the evolutionary models are currently fully sequenced or top-listed for the near future (Table 1).

Zebrafish and medaka have optimal features for conducting large-scale drug screens. For technical reasons, this has been developed for embryonic phenotypes. However, when it comes to adult phenotypes, at least some of the evolutionary models will provide powerful complementary platforms for drug screening.

One of the values of the zebrafish and medaka models is the ease with which transgenic fish can be produced; in addition, target genes can be readily inactivated in these species. In this respect, the evolutionary models are lagging behind. Proof-of-principle evidence has been reported for the turquoise killifish, for which stable GFP-expressing lines were generated (Hartmann and Englert, 2012). Hence, it can be assumed that this technology, as well as the more demanding gene-knockout approaches, will also be possible in the other killifish models, and probably in all freshwater fish species. In other species, such as live-bearing fish (*Xiphophorus* and *Poecilia* species) or the marine models, for which the development of transgenic fish is more complicated, this might not be accomplished in the foreseeable future. However, as previous examples have shown, evolutionary mutant models can be combined with established laboratory models for functional and translational studies, e.g. by expressing the gene of interest from the evolutionary model as a transgene in zebrafish and medaka, or by knocking down the zebrafish or medaka homolog (Schartl et al., 2010; Yergeau et al., 2005).

Another alternative for the evolutionary models might come from the possibility of performing large-scale screens with small-molecule libraries. Compounds that drive a knockdown of a particular protein could be used for functional studies also in those species for which transgenic technologies are not available.

Intuitively, taking the evolutionary models out of the field and doing experiments on laboratory-raised offspring or even established lines might seem to diminish their value, particularly in terms of genetic diversity. However, laboratory lines are useful for the isolation, conservation and in-depth study of certain alleles or allele combinations that are relevant to the development of a disease condition.

A general weakness in the fish field is the minimal availability or even complete lack of cell culture systems. Even for widely used models, only a handful of superficially characterized cell lines exist. The importance of defined *in vitro* systems for in-depth molecular analyses and for complementation of functional studies is paramount. Although there are now several examples where the biochemistry and cellular functions of fish proteins have been studied successfully in mammalian cells, there is a clear need to generate more cell lines from various tissues from the mainstream fish models, and particularly for the evolutionary mutant models.

Progress in the fish field also depends, to some extent, on the availability of experimental reagents. Reagents for analytical and functional studies at the nucleic acid level can now be produced with relative ease for almost any organism by any lab. However, the prime tool for protein studies, the analytical antibodies, are much more demanding to produce and a single research team can generate only a handful of them, at best. The large array of commercially available antibodies for mammalian models, and increasingly also for zebrafish, only evoke envy by those that study other fish species. This important gap will be difficult to seal in the near future, and researchers might have to rely on cross-reacting antibodies from zebrafish in the short term. Sharing reagents and relevant information (as well as other data) can be the tasks of a central fish model database, and should motivate the field to build up a network of researchers that work on non-mainstream models.

The traditional approach in the domesticated models is to generate ‘artificial’ variation in the laboratory by mutagenesis. Nonetheless, natural selection is a potent generator of variation as well. Fish are extremely bio-diverse and make up more than half of the known 60,000 vertebrate species. It could be argued that variation among wild fish species far exceeds the variation that has been generated in the laboratory. The little that we know about almost all those fish species should encourage us to carefully look out for the multitude of novel evolutionary mutant models that surely exist, and that will help to improve our understanding of human diseases, their diagnosis and treatment.

## References

[b1-0070181] Albertson R. C. Kocher T. D. (2006). Genetic and developmental basis of cichlid trophic diversity. Heredity 97, 211–221 16835594 10.1038/sj.hdy.6800864

[b2-0070181] Albertson R. C. Cresko W. Detrich H. W. III Postlethwait J. H. (2009). Evolutionary mutant models for human disease. Trends Genet. 25, 74–81 19108930 10.1016/j.tig.2008.11.006 PMC2828043

[b3-0070181] Albertson R. C. Yan Y. L. Titus T. A. Pisano E. Vacchi M. Yelick P. C. Detrich H. W. III Postlethwait J. H. (2010). Molecular pedomorphism underlies craniofacial skeletal evolution in Antarctic notothenioid fishes. BMC Evol. Biol. 10, 4 20053275 10.1186/1471-2148-10-4 PMC2824663

[b4-0070181] Amatruda J. F. Patton E. E. (2008). Genetic models of cancer in zebrafish. Int Rev Cell Mol Biol 271, 1–34 19081540 10.1016/S1937-6448(08)01201-X

[b5-0070181] Amores A. Force A. Yan Y. L. Joly L. Amemiya C. Fritz A. Ho R. K. Langeland J. Prince V. Wang Y. L. (1998). Zebrafish hox clusters and vertebrate genome evolution. Science 282, 1711–1714 9831563 10.1126/science.282.5394.1711

[b6-0070181] Baudler M. Schartl M. Altschmied J. (1999). Specific activation of a STAT family member in *Xiphophorus* melanoma cells. Exp. Cell Res. 249, 212–220 10366420 10.1006/excr.1999.4470

[b7-0070181] Bolker J. (2012). Model organisms: There’s more to life than rats and flies. Nature 491, 31–33 23128209 10.1038/491031a

[b8-0070181] Borowsky R. Wilkens H. (2002). Mapping a cave fish genome: polygenic systems and regressive evolution. J. Hered. 93, 19–21 12011170 10.1093/jhered/93.1.19

[b9-0070181] Braasch I. Postlethwait J. H. I. (2012). Polyploidy in fish and the teleost genome duplication. In Polyploidy and Genome Evolution (ed. Soltis P. S. Soltis D. E.), pp. 341–383: Springer

[b10-0070181] Burke A. C. Rosa-Molinar E. (2002). Starting from fins: parallelism in the evolution of limbs and genitalia. The fin-to-limb transition. Evol. Dev. 4, 375–377 12356267 10.1046/j.1525-142x.2002.02024.x

[b11-0070181] Burnett K. G. Bain L. J. Baldwin W. S. Callard G. V. Cohen S. Di Giulio R. T. Evans D. H. Gómez-Chiarri M. Hahn M. E. Hoover C. A. (2007). Fundulus as the premier teleost model in environmental biology: opportunities for new insights using genomics. Comp Biochem Physiol 2D, 257–286 10.1016/j.cbd.2007.09.001 PMC2128618 18071578

[b12-0070181] Cirelli C. (2013). Sleep and synaptic changes. Curr. Opin. Neurobiol. 23, 841–846 23623392 10.1016/j.conb.2013.04.001 PMC4552336

[b13-0070181] Cobourne M. T. Green J. B. (2012). Hedgehog signalling in development of the secondary palate. Front. Oral Biol. 16, 52–59 22759669 10.1159/000337543

[b14-0070181] Davis R. H. (2004). The age of model organisms. Nat. Rev. Genet. 5, 69–76 14708017 10.1038/nrg1250

[b15-0070181] Detrich H. W. III Yergeau D. A. (2004). Comparative genomics in erythropoietic gene discovery: synergisms between the Antarctic icefishes and the zebrafish. Methods Cell Biol. 77, 475–503 15602928 10.1016/s0091-679x(04)77026-0

[b16-0070181] Duboué E. R. Keene A. C. Borowsky R. L. (2011). Evolutionary convergence on sleep loss in cavefish populations. Curr. Biol. 21, 671–676 21474315 10.1016/j.cub.2011.03.020

[b17-0070181] Duboué E. R. Borowsky R. L. Keene A. C. (2012). β-adrenergic signaling regulates evolutionarily derived sleep loss in the Mexican cavefish. Brain Behav. Evol. 80, 233–243 22922609 10.1159/000341403

[b18-0070181] Eberhart J. K. He X. Swartz M. E. Yan Y. L. Song H. Boling T. C. Kunerth A. K. Walker M. B. Kimmel C. B. Postlethwait J. H. (2008). MicroRNA Mirn140 modulates Pdgf signaling during palatogenesis. Nat. Genet. 40, 290–298 18264099 10.1038/ng.82 PMC2747601

[b19-0070181] Fan H. (1994). Retroviruses and their role in cancer. In The Retroviridae, Vol. 3 (ed. Levy J. A.), pp. 313–362 New York, NY: Plenum Press

[b20-0070181] Fernandez A. A. Bowser P. R. (2010). Selection for a dominant oncogene and large male size as a risk factor for melanoma in the Xiphophorus animal model. Mol. Ecol. 19, 3114–3123 20618898 10.1111/j.1365-294X.2010.04738.x PMC2911510

[b21-0070181] Fernandez A. A. Paniker L. Garcia R. Mitchell D. L. (2012). Recent advances in sunlight-induced carcinogenesis using the Xiphophorus melanoma model. Comp. Biochem. Physiol. 155, 64–70 10.1016/j.cbpc.2011.03.007 PMC3164944 21457786

[b22-0070181] Force A. Lynch M. Pickett F. B. Amores A. Yan Y. L. Postlethwait J. (1999). Preservation of duplicate genes by complementary, degenerative mutations. Genetics 151, 1531–1545 10101175 10.1093/genetics/151.4.1531 PMC1460548

[b23-0070181] Franck D. Dikomey M. Schartl M. (2001). Selection and the maintenance of a colour pattern polymorphism in the green swordtail (Xiphophorus helleri). Behaviour 138, 467–486

[b24-0070181] Geissinger E. Weisser C. Fischer P. Schartl M. Wellbrock C. (2002). Autocrine stimulation by osteopontin contributes to antiapoptotic signalling of melanocytes in dermal collagen. Cancer Res. 62, 4820–4828 12183442

[b25-0070181] Gordon M. (1927). The genetics of a viviparous top-minnow Platypoecilus: the inheritance of two kinds of melanophores. Genetics 12, 253–283 17246524 10.1093/genetics/12.3.253 PMC1200943

[b26-0070181] Gross J. B. (2012). The complex origin of Astyanax cavefish. BMC Evol. Biol. 12, 105 22747496 10.1186/1471-2148-12-105 PMC3464594

[b27-0070181] Hárosi F. I. von Herbing I. H. Van Keuren J. R. (1998). Sickling of anoxic red blood cells in fish. Biol. Bull. 195, 5–11 9739547 10.2307/1542769

[b28-0070181] Hartmann N. Englert C. (2012). A microinjection protocol for the generation of transgenic killifish (Species: Nothobranchius furzeri). Dev. Dyn. 241, 1133–1141 22473915 10.1002/dvdy.23789

[b29-0070181] Häussler G. (1928). Über melanombildungen bei bastarden von xiphophorus maculatus var. rubra. Klin. Wochenschr. 7, 1561–1562

[b30-0070181] Heston W. E. (1982). Cancer, a Comprehensive Treatise. New York, NY: Plenum Press

[b31-0070181] Hinton D. E. Kullman S. W. Hardman R. C. Volz D. C. Chen P. J. Carney M. Bencic D. C. (2005). Resolving mechanisms of toxicity while pursuing ecotoxicological relevance? Mar. Pollut. Bull. 51, 635–648 16154600 10.1016/j.marpolbul.2005.07.020

[b32-0070181] Horn S. Figl A. Rachakonda P. S. Fischer C. Sucker A. Gast A. Kadel S. Moll I. Nagore E. Hemminki K. (2013). TERT promoter mutations in familial and sporadic melanoma. Science 339, 959–961 23348503 10.1126/science.1230062

[b33-0070181] Howe K. Clark M. D. Torroja C. F. Torrance J. Berthelot C. Muffato M. Collins J. E. Humphray S. McLaren K. Matthews L. (2013). The zebrafish reference genome sequence and its relationship to the human genome. Nature 496, 498–503 23594743 10.1038/nature12111 PMC3703927

[b34-0070181] Hu Q. Gao F. Tian W. Ruteshouser E. C. Wang Y. Lazar A. Stewart J. Strong L. C. Behringer R. R. Huff V. (2011). Wt1 ablation and Igf2 upregulation in mice result in Wilms tumors with elevated ERK1/2 phosphorylation. J. Clin. Invest. 121, 174–183 21123950 10.1172/JCI43772 PMC3007149

[b35-0070181] Huang F. W. Hodis E. Xu M. J. Kryukov G. V. Chin L. Garraway L. A. (2013). Highly recurrent TERT promoter mutations in human melanoma. Science 339, 957–959 23348506 10.1126/science.1229259 PMC4423787

[b36-0070181] Jackson E. W. Wolf H. Sinnhuber R. O. (1968). The relationship of hepatoma in rainbow trout to aflatoxin contamination and cottonseed meal. Cancer Res. 28, 987–991 4297348

[b37-0070181] Jeffery W. R. (2009). Chapter 8. Evolution and development in the cavefish Astyanax. Curr. Top. Dev. Biol. 86, 191–221 19361694 10.1016/S0070-2153(09)01008-4 PMC3594791

[b38-0070181] Jing L. Zon L. I. (2011). Zebrafish as a model for normal and malignant hematopoiesis. Dis. Model. Mech. 4, 433–438 21708900 10.1242/dmm.006791 PMC3124047

[b39-0070181] Kasahara M. Naruse K. Sasaki S. Nakatani Y. Qu W. Ahsan B. Yamada T. Nagayasu Y. Doi K. Kasai Y. (2007). The medaka draft genome and insights into vertebrate genome evolution. Nature 447, 714–719 17554307 10.1038/nature05846

[b40-0070181] Kirschner J. Weber D. Neuschl C. Franke A. Böttger M. Zielke L. Powalsky E. Groth M. Shagin D. Petzold A. (2012). Mapping of quantitative trait loci controlling lifespan in the short-lived fish Nothobranchius furzeri – a new vertebrate model for age research. Aging Cell 11, 252–261 22221414 10.1111/j.1474-9726.2011.00780.x PMC3437503

[b41-0070181] Kluger H. M. Hoyt K. Bacchiocchi A. Mayer T. Kirsch J. Kluger Y. Sznol M. Ariyan S. Molinaro A. Halaban R. (2011). Plasma markers for identifying patients with metastatic melanoma. Clin. Cancer Res. 17, 2417–2425 21487066 10.1158/1078-0432.CCR-10-2402 PMC3415234

[b42-0070181] Kock K.-H. (2005). Antarctic icefishes (Channichthyidae): a unique family of fishes. Polar Biol. 28, 862–895

[b43-0070181] Kosswig C. (1928). Über kreuzungen zwischen den teleostiern xiphophorus helleri und platypoecilus maculatus. Z. Indukt. Abstamm. Vererbungsl. 47, 150–158

[b44-0070181] Lampert K. P. Schartl M. (2008). The origin and evolution of a unisexual hybrid: Poecilia formosa. Philos. Trans. R. Soc. B 363, 2901–2909 10.1098/rstb.2008.0040 PMC2606734 18508756

[b45-0070181] Lampert K. P. Schmidt C. Fischer P. Volff J. N. Hoffmann C. Muck J. Lohse M. J. Ryan M. J. Schartl M. (2010). Determination of onset of sexual maturation and mating behavior by melanocortin receptor 4 polymorphisms. Curr. Biol. 20, 1729–1734 20869245 10.1016/j.cub.2010.08.029

[b46-0070181] Lauderdale J. D. Wilensky J. S. Oliver E. R. Walton D. S. Glaser T. (2000). 3′ deletions cause aniridia by preventing PAX6 gene expression. Proc. Natl. Acad. Sci. USA 97, 13755–13759 11087823 10.1073/pnas.240398797 PMC17648

[b47-0070181] Lettice L. A. Heaney S. J. Purdie L. A. Li L. de Beer P. Oostra B. A. Goode D. Elgar G. Hill R. E. de Graaff E. (2003). A long-range Shh enhancer regulates expression in the developing limb and fin and is associated with preaxial polydactyly. Hum. Mol. Genet. 12, 1725–1735 12837695 10.1093/hmg/ddg180

[b48-0070181] Liao J. B. (2006). Viruses and human cancer. Yale J. Biol. Med. 79, 115–122 17940621 PMC1994798

[b49-0070181] Loots G. G. Kneissel M. Keller H. Baptist M. Chang J. Collette N. M. Ovcharenko D. Plajzer-Frick I. Rubin E. M. (2005). Genomic deletion of a long-range bone enhancer misregulates sclerostin in Van Buchem disease. Genome Res. 15, 928–935 15965026 10.1101/gr.3437105 PMC1172036

[b50-0070181] Masahito P. Ishikawa T. Okamoto N. Sugano H. (1992). Nephroblastomas in the Japanese eel, Anguilla japonica Temminck and Schlegel. Cancer Res. 52, 2575–2579 1314699

[b51-0070181] Meierjohann S. Schartl M. (2006). From Mendelian to molecular genetics: the Xiphophorus melanoma model. Trends Genet. 22, 654–661 17034900 10.1016/j.tig.2006.09.013

[b52-0070181] Meijer A. H. Spaink H. P. (2011). Host-pathogen interactions made transparent with the zebrafish model. Curr. Drug Targets 12, 1000–1017 21366518 10.2174/138945011795677809 PMC3319919

[b53-0070181] Meyer A. Schartl M. (1999). Gene and genome duplications in vertebrates: the one-to-four (-to-eight in fish) rule and the evolution of novel gene functions. Curr. Opin. Cell Biol. 11, 699–704 10600714 10.1016/s0955-0674(99)00039-3

[b54-0070181] Meyle K. D. Guldberg P. (2009). Genetic risk factors for melanoma. Hum. Genet. 126, 499–510 19585149 10.1007/s00439-009-0715-9

[b55-0070181] Mione M. C. Trede N. S. (2010). The zebrafish as a model for cancer. Dis. Model. Mech. 3, 517–523 20354112 10.1242/dmm.004747 PMC2931530

[b56-0070181] Mitchell D. Paniker L. Sanchez G. Trono D. Nairn R. (2007). The etiology of sunlight-induced melanoma in Xiphophorus hybrid fish. Mol. Carcinog. 46, 679–684 17477377 10.1002/mc.20341

[b57-0070181] Morcinek J. C. Weisser C. Geissinger E. Schartl M. Wellbrock C. (2002). Activation of STAT5 triggers proliferation and contributes to anti-apoptotic signalling mediated by the oncogenic Xmrk kinase. Oncogene 21, 1668–1678 11896598 10.1038/sj.onc.1205148

[b58-0070181] Nicholls J. J. Brassill M. J. Williams G. R. Bassett J. H. (2012). The skeletal consequences of thyrotoxicosis. J. Endocrinol. 213, 209–221 22454529 10.1530/JOE-12-0059

[b59-0070181] O’Quin K. E. Yoshizawa M. Doshi P. Jeffery W. R. (2013). Quantitative genetic analysis of retinal degeneration in the blind cavefish Astyanax mexicanus. PLoS ONE 8, e57281 23437360 10.1371/journal.pone.0057281 PMC3577753

[b60-0070181] Patton E. E. Mitchell D. L. Nairn R. S. (2010). Genetic and environmental melanoma models in fish. Pigment Cell Melanoma Res 23, 314–337 20230482 10.1111/j.1755-148X.2010.00693.x PMC2881310

[b61-0070181] Postlethwait J. H. Woods I. G. Ngo-Hazelett P. Yan Y. L. Kelly P. D. Chu F. Huang H. Hill-Force A. Talbot W. S. (2000). Zebrafish comparative genomics and the origins of vertebrate chromosomes. Genome Res. 10, 1890–1902 11116085 10.1101/gr.164800

[b62-0070181] Protas M. E. Hersey C. Kochanek D. Zhou Y. Wilkens H. Jeffery W. R. Zon L. I. Borowsky R. Tabin C. J. (2006). Genetic analysis of cavefish reveals molecular convergence in the evolution of albinism. Nat. Genet. 38, 107–111 16341223 10.1038/ng1700

[b63-0070181] Protas M. Conrad M. Gross J. B. Tabin C. Borowsky R. (2007). Regressive evolution in the Mexican cave tetra, Astyanax mexicanus. Curr. Biol. 17, 452–454 17306543 10.1016/j.cub.2007.01.051 PMC2570642

[b64-0070181] Rahn J. J. Gibbs P. D. Schmale M. C. (2004). Patterns of transcription of a virus-like agent in tumor and non-tumor tissues in bicolor damselfish. Comp. Biochem. Physiol. 138C, 401–409 10.1016/j.cca.2004.06.008 15533798

[b65-0070181] Renshaw S. A. Trede N. S. (2012). A model 450 million years in the making: zebrafish and vertebrate immunity. Dis. Model. Mech. 5, 38–47 22228790 10.1242/dmm.007138 PMC3255542

[b66-0070181] Roberts R. B. Hu Y. Albertson R. C. Kocher T. D. (2011). Craniofacial divergence and ongoing adaptation via the hedgehog pathway. Proc. Natl. Acad. Sci. USA 108, 13194–13199 21788496 10.1073/pnas.1018456108 PMC3156204

[b67-0070181] Sabherwal N. Bangs F. Röth R. Weiss B. Jantz K. Tiecke E. Hinkel G. K. Spaich C. Hauffa B. P. van der Kamp H. (2007). Long-range conserved non-coding SHOX sequences regulate expression in developing chicken limb and are associated with short stature phenotypes in human patients. Hum. Mol. Genet. 16, 210–222 17200153 10.1093/hmg/ddl470

[b68-0070181] Santoriello C. Zon L. I. (2012). Hooked! Modeling human disease in zebrafish. J. Clin. Invest. 122, 2337–2343 22751109 10.1172/JCI60434 PMC3386812

[b69-0070181] Sbaihi M. Kacem A. Aroua S. Baloche S. Rousseau K. Lopez E. Meunier F. Dufour S. (2007). Thyroid hormone-induced demineralisation of the vertebral skeleton of the eel, Anguilla anguilla. Gen. Comp. Endocrinol. 151, 98–107 17280664 10.1016/j.ygcen.2006.12.009

[b70-0070181] Schartl M. (2008). Evolution of Xmrk: an oncogene, but also a speciation gene? Bioessays 30, 822–832 18693261 10.1002/bies.20807

[b71-0070181] Schartl A. Malitschek B. Kazianis S. Borowsky R. Schartl M. (1995). Spontaneous melanoma formation in nonhybrid *Xiphophorus*. Cancer Res. 55, 159–165 7805027

[b72-0070181] Schartl A. Hornung U. Nanda I. Wacker R. Müller-Hermelink H. K. Schlupp I. Parzefall J. Schmid M. Schartl M. (1997). Susceptibility to the development of pigment cell tumors in a clone of the Amazon molly, Poecilia formosa, introduced through a microchromosome. Cancer Res. 57, 2993–3000 9230214

[b73-0070181] Schartl M. Wilde B. Laisney J. A. Taniguchi Y. Takeda S. Meierjohann S. (2010). A mutated EGFR is sufficient to induce malignant melanoma with genetic background-dependent histopathologies. J. Invest. Dermatol. 130, 249–258 19609310 10.1038/jid.2009.213

[b74-0070181] Schartl M. Kneitz S. Wilde B. Wagner T. Henkel C. V. Spaink H. P. Meierjohann S. (2012). Conserved expression signatures between medaka and human pigment cell tumors. PLoS ONE 7, e37880 22693581 10.1371/journal.pone.0037880 PMC3365055

[b75-0070181] Schartl M. Walter R. B. Shen Y. Garcia T. Catchen J. Amores A. Braasch I. Chalopin D. Volff J.-N. Lesch K.-P. (2013). The genome of the platyfish, Xiphophorus maculatus, provides insights into evolutionary adaptation and several complex traits. Nat. Genet. 45, 567–572 23542700 10.1038/ng.2604 PMC3677569

[b76-0070181] Schmale M. C. Hensley G. Udey L. R. (1983). Neurofibromatosis, von Recklinghausen’s disease, multiple schwannomas, malignant schwannomas. Multiple schwannomas in the bicolor damselfish, Pomacentrus partitus (pisces, pomacentridae). Am. J. Pathol. 112, 238–241 6410922 PMC1916251

[b77-0070181] Schmale M. C. Hensley G. T. Udey L. R. (1986). Neurofibromatosis in the bicolor damselfish (Pomacentrus partitus) as a model of von Recklinghausen neurofibromatosis. Ann. N. Y. Acad. Sci. 486, 386–402 3105403 10.1111/j.1749-6632.1986.tb48092.x

[b78-0070181] Sidell B. D. O’Brien K. M. (2006). When bad things happen to good fish: the loss of hemoglobin and myoglobin expression in Antarctic icefishes. J. Exp. Biol. 209, 1791–1802 16651546 10.1242/jeb.02091

[b79-0070181] Spivakov M. Auer T. O. Peravali R. Dunham I. Dolle D. Fujiyama A. Toyoda A. Aizu T. Minakuchi Y. Loosli F. (2013). Genomic and Phenotypic Characterisation of a Wild Medaka Population: Establishing an Isogenic Population Genetic Resource in Fish. arXiv:1304.4515. [q-bio.GN] 10.1534/g3.113.008722 PMC3962483 24408034

[b80-0070181] Stöck M. Lampert K. P. Möller D. Schlupp I. Schartl M. (2010). Monophyletic origin of multiple clonal lineages in an asexual fish (Poecilia formosa). Mol. Ecol. 19, 5204–5215 20964758 10.1111/j.1365-294X.2010.04869.x

[b81-0070181] Strecker U. Bernatchez L. Wilkens H. (2003). Genetic divergence between cave and surface populations of Astyanax in Mexico (Characidae, Teleostei). Mol. Ecol. 12, 699–710 12675825 10.1046/j.1365-294x.2003.01753.x

[b82-0070181] Terzibasi E. Valenzano D. R. Cellerino A. (2007). The short-lived fish Nothobranchius furzeri as a new model system for aging studies. Exp. Gerontol. 42, 81–89 17049789 10.1016/j.exger.2006.06.039

[b83-0070181] Tobler M. Schlupp I. (2005). Parasites in sexual and asexual mollies (Poecilia, Poeciliidae, Teleostei): a case for the Red Queen? Biol. Lett. 1, 166–168 17148156 10.1098/rsbl.2005.0305 PMC1626213

[b84-0070181] Velagaleti G. V. Bien-Willner G. A. Northup J. K. Lockhart L. H. Hawkins J. C. Jalal S. M. Withers M. Lupski J. R. Stankiewicz P. (2005). Position effects due to chromosome breakpoints that map approximately 900 Kb upstream and approximately 1.3 Mb downstream of SOX9 in two patients with campomelic dysplasia. Am. J. Hum. Genet. 76, 652–662 15726498 10.1086/429252 PMC1199302

[b85-0070181] Walsh P. J. Mensinger A. F. Highstein S. M. (2008). Toadfish as biomedical models. Oceans and Human Health: Risks and Remedies from the Seas. Amsterdam: Academic Press

[b86-0070181] Walter R. B. Kazianis S. (2001). Xiphophorus interspecies hybrids as genetic models of induced neoplasia. ILAR J. 42, 299–321 11581522 10.1093/ilar.42.4.299

[b87-0070181] Wellbrock C. Schartl M. (1999). Multiple binding sites in the growth factor receptor Xmrk mediate binding to p59fyn, GRB2 and Shc. Eur. J. Biochem. 260, 275–283 10091608 10.1046/j.1432-1327.1999.00180.x

[b88-0070181] Wellbrock C. Schartl M. (2000). Activation of phosphatidylinositol 3-kinase by a complex of p59fyn and the receptor tyrosine kinase Xmrk is involved in malignant transformation of pigment cells. Eur. J. Biochem. 267, 3513–3522 10848967 10.1046/j.1432-1327.2000.01378.x

[b89-0070181] Wellbrock C. Weisser C. Hassel J. C. Fischer P. Becker J. Vetter C. S. Behrmann I. Kortylewski M. Heinrich P. C. Schartl M. (2005). STAT5 contributes to interferon resistance of melanoma cells. Curr. Biol. 15, 1629–1639 16169484 10.1016/j.cub.2005.08.036

[b90-0070181] Wilkens H. (1988). Evolution and genetics of epigean and cave Astyanax fasciatus (Characidae, Pisces). Evol. Biol. 23, 271–367

[b91-0070181] Wilkens H. (2010). Genes, modules and the evolution of cave fish. Heredity 105, 413–422 20068586 10.1038/hdy.2009.184

[b92-0070181] Williams D. E. (2012). The rainbow trout liver cancer model: response to environmental chemicals and studies on promotion and chemoprevention. Comp Biochem Physiol 155C, 121–127 10.1016/j.cbpc.2011.05.013 PMC3219792 21704190

[b93-0070181] Wittbrodt J. Shima A. Schartl M. (2002). Medaka – a model organism from the far East. Nat. Rev. Genet. 3, 53–64 11823791 10.1038/nrg704

[b94-0070181] Woodhead A. D. Setlow R. B. Pond V. (1984). The Amazon molly, Poecilia formosa, as a test animal in carcinogenicity studies: chronic exposures to physical agents. Natl. Cancer Inst. Monogr. 65, 45–52 6087147

[b95-0070181] Yergeau D. A. Cornell C. N. Parker S. K. Zhou Y. Detrich H. W. III (2005). bloodthirsty, an RBCC/TRIM gene required for erythropoiesis in zebrafish. Dev. Biol. 283, 97–112 15890331 10.1016/j.ydbio.2005.04.006

[b96-0070181] Young N. M. Chong H. J. Hu D. Hallgrímsson B. Marcucio R. S. (2010). Quantitative analyses link modulation of sonic hedgehog signaling to continuous variation in facial growth and shape. Development 137, 3405–3409 20826528 10.1242/dev.052340 PMC2947484

[b97-0070181] Zhou Y. Dai D. L. Martinka M. Su M. Zhang Y. Campos E. I. Dorocicz I. Tang L. Huntsman D. Nelson C. (2005). Osteopontin expression correlates with melanoma invasion. J. Invest. Dermatol. 124, 1044–1052 15854047 10.1111/j.0022-202X.2005.23680.x

[b98-0070181] Zon L. I. Peterson R. T. (2005). In vivo drug discovery in the zebrafish. Nat. Rev. Drug Discov. 4, 35–44 15688071 10.1038/nrd1606

